# Access Point Selection Game with Mobile Users Using Correlated Equilibrium

**DOI:** 10.1371/journal.pone.0116592

**Published:** 2015-03-18

**Authors:** Insoo Sohn

**Affiliations:** Division of Electronics & Electrical Engineering, Dongguk University—Seoul, Seoul, Republic of Korea; Tianjin University of Technology, CHINA

## Abstract

One of the most important issues in wireless local area network (WLAN) systems with multiple access points (APs) is the AP selection problem. Game theory is a mathematical tool used to analyze the interactions in multiplayer systems and has been applied to various problems in wireless networks. Correlated equilibrium (CE) is one of the powerful game theory solution concepts, which is more general than the Nash equilibrium for analyzing the interactions in multiplayer mixed strategy games. A game-theoretic formulation of the AP selection problem with mobile users is presented using a novel scheme based on a regret-based learning procedure. Through convergence analysis, we show that the joint actions based on the proposed algorithm achieve CE. Simulation results illustrate that the proposed algorithm is effective in a realistic WLAN environment with user mobility and achieves maximum system throughput based on the game-theoretic formulation.

## Introduction

One of the most important issues in wireless local area network (WLAN) systems with multiple access points (Aps) is the AP selection problem [1–2]. The performance experienced by WLAN users depends strongly on the appropriate selection of APs that will offer the best services. A simple AP selection algorithm that selects an AP based only on the received signal strength can lead to imbalanced traffic load in APs and can result in degradation of the network performance.

Game theory is a mathematical tool used to analyze the interactions in multiplayer systems [3–6] and has been applied to various problems in wireless networks [7–11]. Therefore, we focus on developing a decentralized AP selection algorithm that can enable users to select appropriate APs independently and can maximize user throughput based on a game-theoretic formulation. In the AP selection game, the set of players corresponds to the mobile users, and the set of strategies available to each player is defined by the multiple APs available in the network. Furthermore, all the users are assumed to be mobile, meaning that the users are motivated to move to a different location if user throughput improvement is possible. Therefore, the cost of user mobility must be included in the utility function of the AP selection game model.

In [12], the authors modelled the AP selection problem with mobile users as a game and analyzed the resulting Nash equilibrium (NE) based on a simple myopic algorithm. It was shown that NE can be achieved in a WLAN system with multiple APs consisting of homogeneous users with mobility and a simple arrival/exit model. To enhance the analysis of the AP selection problem based on the game-theoretic formulation, the inclusion of accurate wireless physical channel characteristics and user throughput criteria is desired. Furthermore, NE does not always lead to the best performance in a distributed and competitive multiplayer environment.

Correlated equilibrium (CE) is a generalization of NE based on a joint probability distribution, thus enabling the strategy profile to be correlated arbitrarily [13–14]. Furthermore, CE has a computational advantage over NE because it is guaranteed that a CE solution can be obtained in polynomial time for any game [15–17]. Therefore, CE is a better solution compared to the NE, suited to the design of a distributed algorithm for the AP selection game. Among many approaches for achieving a CE in a given game, a regret-matching procedure is one of the most popular and simplest methods used. A regret-matching procedure is an adaptive strategic learning method based on a criterion defined by past patterns of payoffs [18–20]. Hart and Mas-Collel proved in [14] that in general games, a regret-matching procedure converges into the set of coarse CE.

In this paper, we formulate the AP selection problem in sequential and dynamic user arrival/exit environments as a mixed strategic game with the goal of system throughput optimization. We then propose a simple distributed algorithm for the AP selection game based on the regret-matching procedure. Numerical results are used to highlight the effectiveness of the proposed method in terms of fast convergence and throughput performance.

The remainder of the paper is structured as follows. Section 2 describes the sequential and dynamic user arrival/exit model and the AP selection problem as formulated based on the game theory. In section 3, we propose the distributed AP selection algorithm based on the regret-matching procedure with convergence analysis. The numerical results are presented in section 4, and conclusions are drawn in Section 5.

## System Model

### Sequential User Arrival/Exit Model

We first consider the AP selection problem with simple sequential user arrival and exit pattern in the WLAN system. Let *K* denote the total number of users existing in the network and *K* ∈ *k* = {1, 2, …, *K*} denote the user index. We denote *M* as the total number of APs existing in the network, and *M* ∈ *m* = {1, 2, …, *M*} denotes the AP index. Each user *k* enters the system and selects an AP that maximizes the user throughput based on the current location and the load level status of the APs. The user arrival timing is modeled to be sequential and to complete the AP selection process before the next user enters the system. The users may choose to move to a new AP and reassociate whenever an increase in the user throughput is possible. In this AP selection model, we assume that all users exit the system separated by very short intervals. The main goal of the users is achieved by selecting APs that minimize the traveling distance and have a minimum number of associated users.

### Dynamic User Arrival/Exit Model

We next consider the case of a dynamic user arrival and exit model. In the previous section, we have assumed that all the users arrive sequentially and exit the system almost simultaneously. However, in reality, the user arrival and exit pattern is dynamic and may affect the total user throughput and distance traveled in a complex manner due to continuous variations in real time user distributions. Therefore, to analyze the AP selection performance in a realistic user arrival and exit setting, we assume that the users’ arrival times follow a Poisson distribution with a mean inter-arrival time λ, as shown below

$$f\left(k;\lambda \right)=\frac{\lambda^{k}e^{-\lambda }}{k!}.$$ (1)

Furthermore, the time spent in the system after the arrival follows an exponential distribution with mean in-system time β, as shown below

$$f(x;\beta )=\left\{ \begin{array}{ll} \beta^{-1}e^{-x/\beta } & x\geq 0,\\ 0 & x<0. \end{array}\right.$$ (2)

Thus, user arrival and exit actions will overlap until the completion of all user arrivals. After the completion of all user arrivals, the AP selection process will continue with only user exit actions remaining and will be completed when none of the *K* users are left in the WLAN system. In [21], Barabási has shown that many human action intervals deviate from the conventional Poisson process model and are better approximated by bursty heavy tailed inter-event dynamics with a power law distribution. Therefore, in addition to the Poisson process-based dynamic model, we also consider the power law distribution shown below for dynamic user AP selection analysis.

$$P[X>x]\propto x^{-\alpha }$$ (3)

where the exponent or scaling parameter typically lies in the range 1 < α < 3.

### Strongest-signal-first Method with User Movement

**Fig 1:**
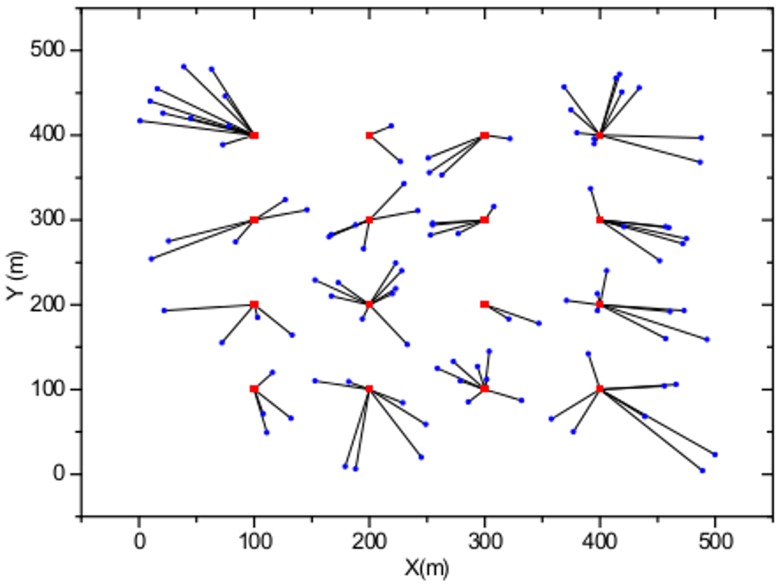
WLAN model with user movement trajectories with uniform user distribution.

**Fig 2:**
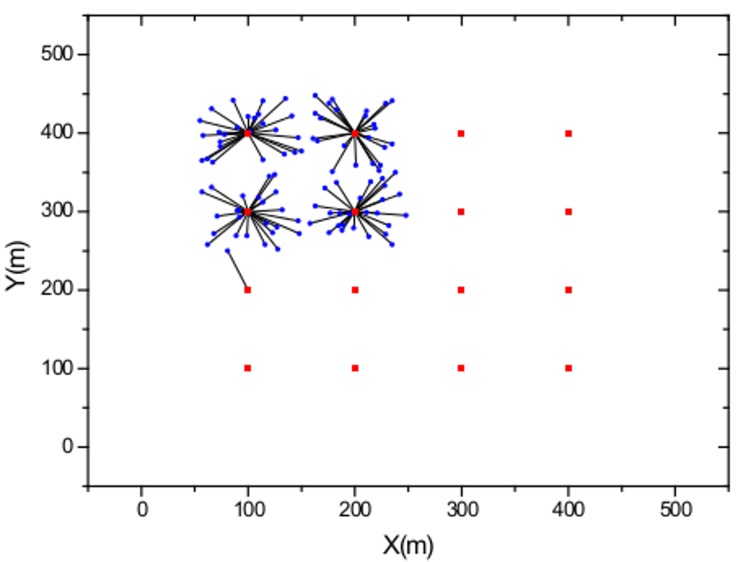
WLAN model with user movement trajectories with non-uniform user distribution.

In this section, we study the user-AP association behavior using the conventional strongest-signal-first (SSF) method, which always associates with users with the strongest received signal strength. Fig. 1 shows the user movement based on the SSF method with *M* = 16 APs distributed evenly with a separating distance of 100 m and a number of users *K* = 100 that are distributed randomly with a uniform distribution. The APs are represented by red squares, and the users are represented by blue circles. As shown in Fig. 1, the users simply associate with APs with the minimum distance relative to the user. Fig. 2 shows the user-AP association behavior with a non-uniform user distribution. Note that based on the SSF method, imbalanced traffic load and degradation in the network performance are expected in this sample scenario.

### Game-Theoretic Model

We now formulate the AP selection problem based on the game-theoretic approach such that the total user throughput is maximized taking the cost of user mobility into account. Formally, the AP selection problem can be formulated as a mixed strategic game that can be expressed as

$$\Gamma =\langle K,{\left\{S_{k}\right\}}_{k\in K},{\left\{u_{k}\right\}}_{k\in K}\rangle ,$$ (4)

where *K* is the set of players corresponding to the mobile users, *S*_*k*_ is the set of strategies for player *k* corresponding to the candidate APs to be associated with, and *u*_*k*_ is the utility function. The goal of the mixed strategic game is to obtain a set of probability distribution Δ(*S*) on *S* that maximizes the expected payoff of all the players. The utility function is a key measure of performance that impacts how the players choose the strategies. In our case, the utility function is designed to measure the maximal average throughput that can be achieved by a user *k* with the cost of distance travelled when AP *m* is selected. We define the utility function for player *i* as

$$u_{k}(s_{k},s_{-k})=\alpha \theta_{k}(s_{k},s_{-k})-d_{k}(s_{k},s_{-k}),$$ (5)

where *s*_-*k*_ is the joint strategy of other players, *θ*_*k*_ (*s*_*k*_, *s*_-*k*_) is the downlink throughput achieved for the selected strategy *s*_*k*_, *d*_*k*_ (*s*_*k*_, *s*_-*k*_) is the distance travelled based on the strategy *s*_*k*_, and *α* is the weight factor for balancing the throughput gain and the distance cost. The throughput of user *k* associated with AP *m* can be expressed as

$$\theta_{k,m}=R_{m}\frac{f(\gamma_{k,m})}{L_{m}^{}},$$ (6)

where *R*_*m*_ denotes the transmission rate of AP *m*; *L*_*m*_ is the number of users associated with AP *m*; and *γ*_*k*,*m*_ is the received signal-to-interference-plus-noise ratio (SINR) of user *k* associated with AP *m*, which can be expressed as

$$\gamma_{k,m}=\frac{p_{m}|h_{k,m}{|}^{2}}{\sigma^{2}+\sum_{n\neq m}p_{n}|h_{k,n}{|}^{2}},$$ (7)

where *p*_*m*_ is the power transmitted by AP *m*, σ^2^ is the noise power, and *h*_*k*,*m*_ is the channel gain between the user *k* and AP *m*. Furthermore, *f*(γ_*k*,*m*_) is the efficiency function approximating the probability of error free packet reception, which can be expressed as

$$f(\gamma_{k,m})={\left(1-e^{-\gamma_{k,m}}\right)}^{P},$$ (8)

where *P* denotes the number of bits in each transmitted data packet.

## Proposed Algorithm

### Regret Matching

We now introduce the correlated equilibrium (CE) solution of the AP selection game, which can be obtained using the regret-matching procedure. In contrast to the Nash equilibrium (NE) solution, which is based on the marginal probability distribution of players’ strategies, CE encourages the selfish players to coordinate their actions based on a joint probability distribution of *N*-tuples of actions that can be viewed as the distribution of recommendations given to the players by some referee. The definition of CE is given below and can interpreted as the joint probability distribution *q* is defined to be a correlated equilibrium if no player *k* can choose a strategy *ś*_*k*_ instead of *s*_*k*_ resulting in higher payoff.

**Definition 1**. A correlated strategy *q* ∈ Δ(*S*) is a CE of Г if and only if, for all *k* ∈ *K* and *(s*_*k*_, *s*_-*k*_) ∈ *S*,

$$\sum_{s_{-k}\in S_{-k}}q(s_{k},s_{-k})u\left(s_{k},s_{-k}\right)\geq \sum_{s_{-k}\in S_{-k}}q(s_{k},s_{-k})u\left(s_{k}^{\prime },s_{-k}\right),$$ (9)

for all *ś*_-*k*_ ∈ *S*_*k*_.

A regret-matching procedure is an important strategic learning method based on a criterion of past patterns of payoffs. The key idea of the regret-matching procedure is to adjust the players’ strategies probabilistically by evaluating the average regret from not having chosen strategy *n* instead of strategy *m* that was selected in the past. The general regret-matching procedure is summarized as follows.

1. Initialization
2. *Utility update*: Calculate the average utility based on the past and present utility payoffs through time *t*_*0*_ as
  $${\bar{u}}_{k,m}^{t_{0}}=\frac{1}{t_{0}}\sum_{t=1}^{t_{0}}u_{k,m}^{t},$$ (10)
3. *Regret update*: Calculate the average regret from not having played the strategy *n* up to time *t*_*0*_ as
  $$\gamma_{n}^{t_{0}}={\bar{u}}_{k,n}^{t_{0}}-{\bar{u}}_{k,m}^{t_{0}},$$ (11)
4. *Strategy update*: Adjust the user strategy based on a probability proportional to the nonnegative part of the player’s regret up through time *t*_*0*._

### Algorithm Description

**Fig 3:**
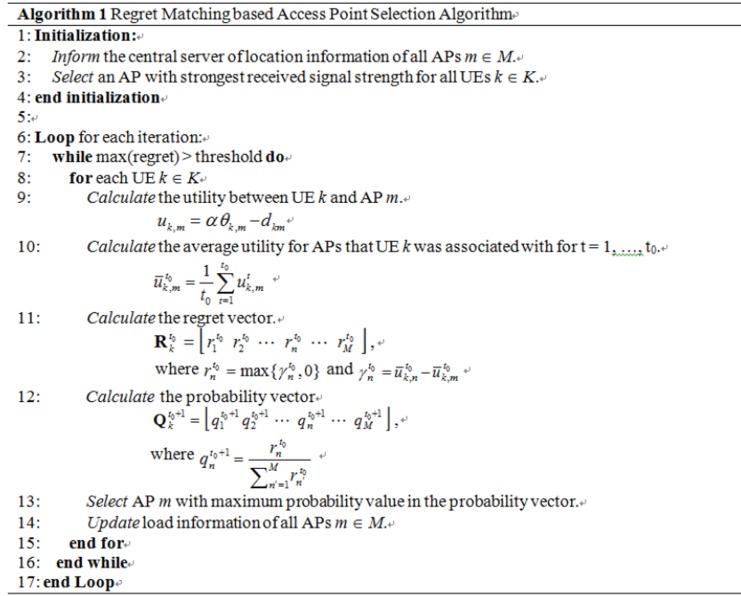
Algorithm 1 Regret-Matching-based Access Point Selection Algorithm

We propose an AP selection algorithm based on the regret-matching procedure presented in the previous section. The details of the algorithm are given in Fig. 3 and can be summarized as follows. First, AP related parameters such as location information is initialized and reported to the central server. Next, each user *k* initially selects an AP with the strongest signal strength as it enters the WLAN system. The main iteration of the algorithm is activated whenever a new user enters the system or a user exits the system. The existing users in the system update the current AP selection strategy based on the calculated regret vector and the probability vector. The iteration stops when the maximum regret value is smaller than the predefined threshold, indicating convergence to CE. The load information of all the APs is updated on every iteration.

### Convergence Analysis

Hart and Mas-Collel proved in [14] that a necessary and sufficient condition for the empirical distribution to converge to the set of correlated equilibrium is that all the regrets converge to zero when the game is played according to the regret-matching procedure by applying Blackwell's approachability theorem. Because the proposed algorithm follows the regret-matching procedure, the convergence to the set of coarse CE is guaranteed and is stated in the following theorem.

**Theorem 1**. If all the users follow the proposed AP selection algorithm, the empirical distribution of joint actions converges almost surely to the set of coarse CE.

**Fig 4:**
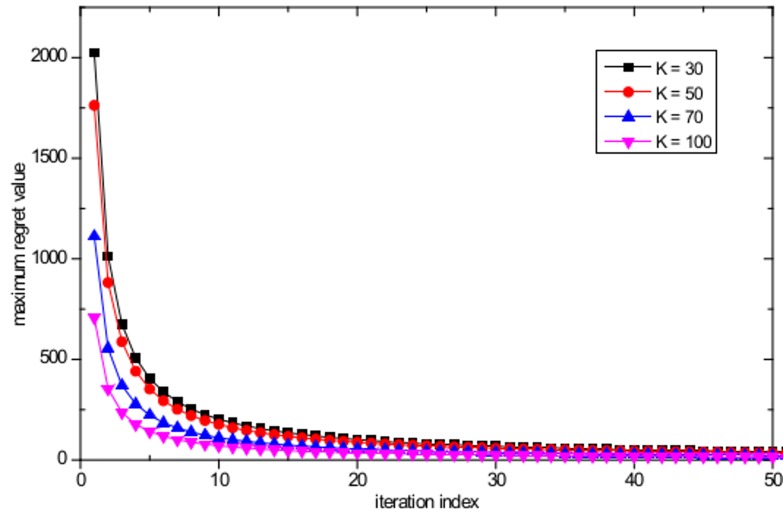
Evolution of maximum regret value of the worst player with *K* = 30, *K* = 50, *K* = 70, and *K* = 100 with uniform user distribution.

**Fig 5:**
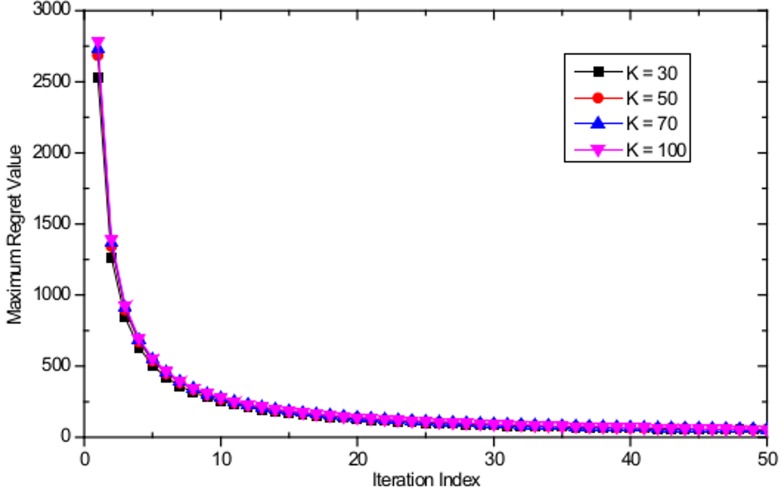
Evolution of maximum regret value of the worst player with *K* = 30, *K* = 50, *K* = 70, and *K* = 100 with non-uniform user distribution.

In this section, the CE convergence of the proposed algorithm is evaluated for both the case of uniform user distribution and non-uniform user distribution in a WLAN with *M* = 16 APs distributed evenly with a separation distance of 100 m and different numbers of users. Fig. 4 shows the evolution of the maximum regret value of the worst player with a uniform user distribution, based on the proposed algorithm, with maximum number of users equal to *K* = 30, *K* = 50, *K* = 70, and *K* = 100. One can note from the figure that regardless of the number of players, all the regret values converge to zero within a few iterations. Fig. 5 shows the evolution of the maximum regret value of the worst player with a non-uniform user distribution with maximum number of users equal to *K* = 30, *K* = 50, *K* = 70, and *K* = 100; similar rapid convergence behavior is observed as in the case of uniform user distribution. Therefore, we can conclude that the joint actions based on the proposed algorithm converge to the CE as stated in Theorem 1.

## Performance Evaluation

### Simulation Environment

The proposed AP selection algorithm is evaluated by studying the total user throughput and the average user distance traveled in the WLAN with various user and AP location patterns and various user arrival and exit models. The APs operate with equal fixed transmit power *p*_*m*_ = 100 mW, the Gaussian noise power *σ* ^*2*^ is 10^–9^ mW, the carrier frequency *f*_*c*_ is 2.4 GHz, the transmission rate is assumed to be the same for all the APs with *R*_*m*_ = 10 Mbps, and the number of bits in each transmitted data packet *P* is set to 10^4^ bits. Furthermore, the weight factor α is given the value of 300, and the number of convergence iteration is set to 30 in all simulations.

### Sequential User Arrival/Exit Environment

**Fig 6:**
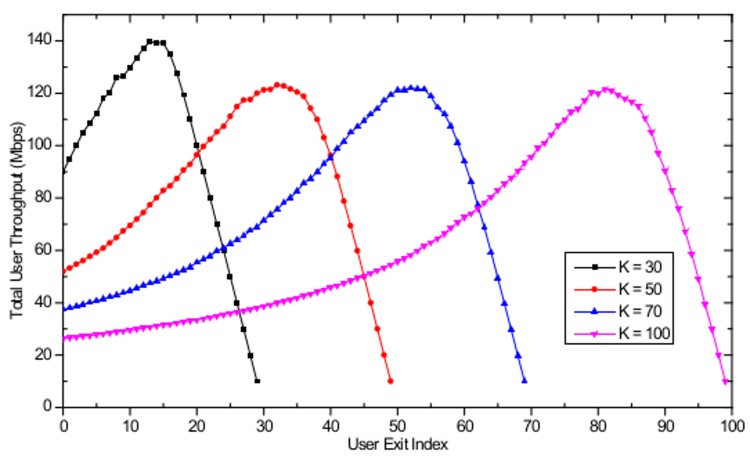
Evolution of total user throughput with user exit index with *K* = 30, *K* = 50, *K* = 70, and *K* = 100.

**Fig 7:**
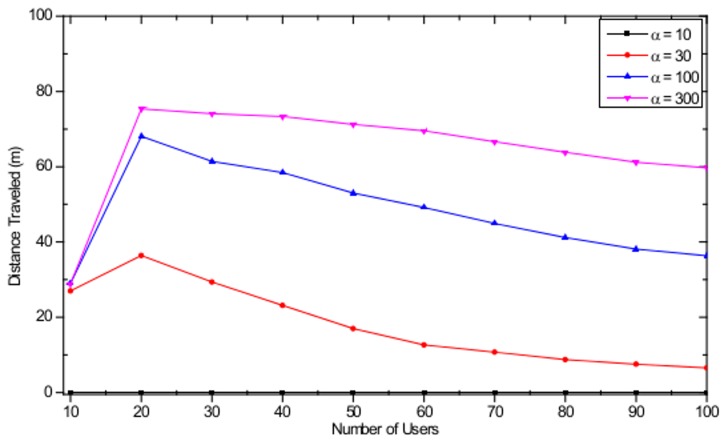
Distance travelled with different number of users with α = 10, α = 30, α = 100, and α = 300.

In the simulations, the number of APs is set to *M* = 16 with a separation distance of 100 m in a 500 m × 500 m WLAN system as shown in Fig. 1. The user arrival timing is modeled to be sequential and to complete the AP selection process before the next user enters the system. We assume that all users exit the system within a very short period of time from each other. Fig. 6 shows the total user throughput evolution with different maximum number of users, defined as the total sum of all *k* users' serviced throughput currently located in the WLAN, starting from the last user arrival at the user arrival stage and ending when the last user exits the system at the user departure stage. As shown in the figure, when the user exit index is equal to 0, which corresponds to the state of user arrival completion, total user throughput of 90 Mbps, 52 Mbps, 37 Mbps, and 26 Mbps are observed for *K* = 30, *K* = 50, *K* = 70, and *K* = 100, respectively. With the sequential user exit, it is observed that the maximum total user throughput is obtained when the exit user index is equal to 14 and 84, for *K* = 30 and *K* = 100, respectively. This phenomenon occurs because that is when the number of users associated with each AP becomes 1, thus fully utilizing the available resources at the AP. After reaching the peak throughput point, graceful performance degradation is observed until only one user is left in the WLAN. From Fig. 6, one can observe that the users are able to appropriately respond to the continuous change in the AP load distribution, due to the user exit, and achieve an optimum performance. This is made possible by selecting an AP selection strategy satisfying the CE conditions for every user exit, based on the regret-matching procedure. Fig. 7 shows the distance traveled by the users from the beginning of the arrival stage to the end of the departure stage in the WLAN with different values of the weight factor α for different maximum number of users *K*. With the increasing value of α, users travel larger distances to obtain maximum throughput. Notice that when α = 10, the users stay with the initial associated APs and do not move at all. Thus, for small α values, the final AP load distribution may be highly imbalanced, resulting in poor user throughput performances.

### Dynamic User Arrival/Exit Environment

**Fig 8:**
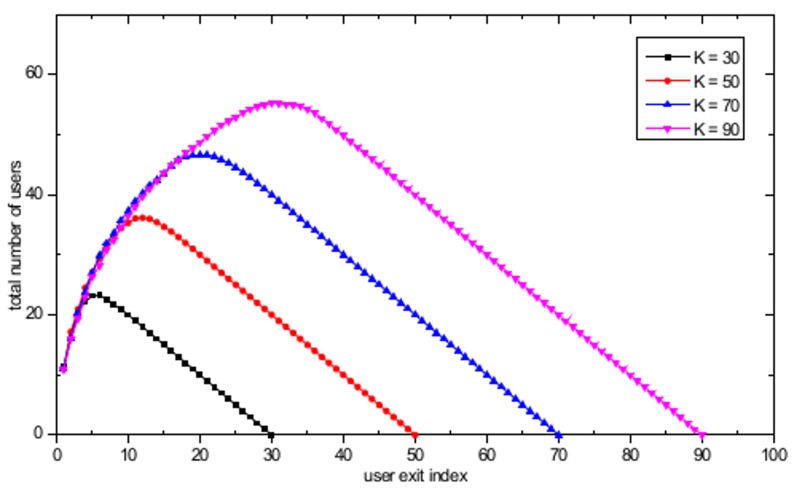
Evolution of total number of users with user exit index with *K* = 30, *K* = 50, *K* = 70, and *K* = 90 based on Poisson Process model.

**Fig 9:**
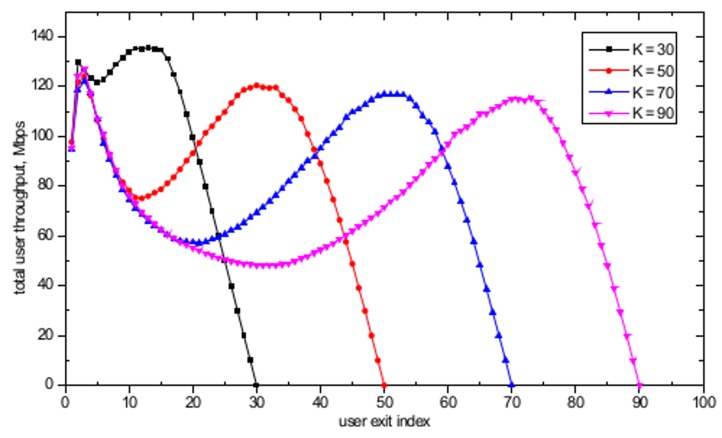
Evolution of total user throughput with user exit index with *K* = 30, *K* = 50, *K* = 70, and *K* = 90 based on Poisson Process model.

**Fig 10:**
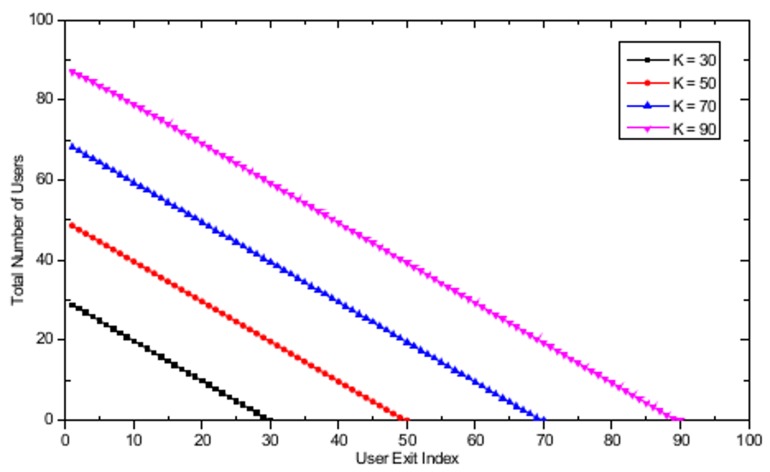
Evolution of total number of users with user exit index with *K* = 30, *K* = 50, *K* = 70, and *K* = 90 based on power law model.

**Fig 11:**
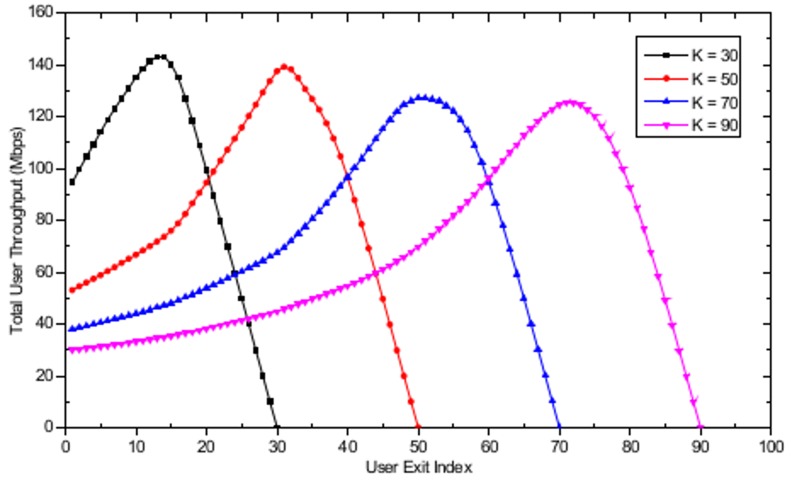
Evolution of total user throughput with user exit index with *K* = 30, *K* = 50, *K* = 70, and *K* = 90 based on power law model.

In the dynamic user arrival/exit simulations, the number of APs is set to *M* = 16 and the APs are randomly distributed in a 500 m × 500 m WLAN system. A realistic user arrival and exit environment is simulated by assuming that the users’ arrival time follows a Poisson distribution with a mean inter-arrival time λ equal to 3 seconds and that the time spent in the system after the arrival follows an exponential distribution with mean in-system time β equal to 300 seconds. Fig. 8 shows the evolution of the total number of users in the WLAN system with the user exit index. For *K* = 70, as the number of users entering the WLAN system increases, the total number of users in the system also increases with the increasing user exit index until 20 users have left the system, as illustrated in Fig. 8. When the user exit index has reached 20, the arrival stage will be completed, and only user exit actions will remain, resulting in a decrease in the total number of users until no users are left in the system. Similar behavior is observed for all other cases. Fig. 9 shows the total user throughput evolution with different maximum number of users, defined as the total sum of all *k* users' serviced throughput currently located in the WLAN with dynamic user arrivals and exits. In Fig. 9, a decrease in total user throughput is observed with the increase in the total number of users in the WLAN system. The decrease in the total user throughput stops when the user exit index is equal to 20 and 30 for *K* = 70 and *K* = 90, respectively. The reason for this cessation is that those are the points in time when all the user arrival actions are completed and only user exit actions remain, thus resulting in increasing total user throughput. Furthermore, it is observed that the maximum total user throughput is obtained when the exit user index is equal to 54 and 74, for *K* = 70 and *K* = 90, respectively. This result is observed because these values cause the number of users associated at each AP to become 1, thus fully utilizing the available resources at the AP. After reaching the peak throughput point, graceful performance degradation is observed until no user is left in the WLAN. Finally, note that there is a sudden increase in the total user throughput between the user exit index equal to 1 and 2. This phenomenon can be explained by observing that the total number of users in the WLAN system increases from 11 to 16 for user exit index equal to 1 and 2, respectively, as shown in Fig. 8. Thus, a sharp increase in the total user throughput is possible by assigning the 16 users evenly to each 16 APs, resulting in efficient resource utilization through the use of the proposed algorithm. Further analysis in the dynamic user arrival and exit environment is performed by assuming that the inter-event time has a power law tail with exponent parameter α equal to 2. The mean event time was chosen to be equal to the Poisson process’s mean event time used in the previous simulations. Fig. 10 shows the evolution of total number of users in the WLAN system with a user exit index based power law model. In contrast to the Poisson process environment based results shown in Fig. 8, only decreasing behavior in total number of users is observed. The apparent reason for this behavior is that due to the burst activity pattern produced by the power law distribution, all the users exit the system within a short period of time from each other. Fig. 11 shows the total user throughput evolution with different maximum number of users with power law distribution. As shown in the figure, the maximum throughput is obtained when the number of users associated with each AP becomes one, as in the Poisson process based dynamic user arrival and exit scenario.

## Conclusions

In this paper, the problem of AP selection in WLAN systems was formulated as a mixed strategic game with a utility function balancing the user throughput and mobility. Furthermore, we proposed a novel distributed AP scheme based on the regret-matching procedure. The proposed method was designed to select an appropriate AP for each user with the goal of maximizing the user throughput with balanced load based on a game- theoretic formulation. Through convergence analysis, we have shown that the joint actions based on the proposed algorithm achieve CE. Simulation results have shown that the proposed scheme is effective in a realistic WLAN environment with user mobility.
